# Benralizumab in bronchiectasis: results from the phase 3 MAHALE study

**DOI:** 10.1183/23120541.01529-2025

**Published:** 2026-08-24

**Authors:** James D. Chalmers, Stefano Aliberti, Lucy Burr, Ulla Møller Weinreich, Miguel Ángel Bergna, Annika Åstrand, Prashanth Basavanna, Ying Fan, Maura Gatti, Calvin N. Ho, Stefan Ivanov, Christopher McCrae, Ana Román, Svitlana Salemio, Maria Jison

**Affiliations:** 1 Department of Respiratory Medicine and Gastroenterology, University of Dundee, Ninewells Hospital and Medical School, Dundee, UK; 2 Radcliffe Department of Medicine, University of Oxford, Oxford, UK; 3 Department of Biomedical Sciences, Humanitas University, Milan, Italy; 4 IRCCS Humanitas Research Hospital, Milan, Italy; 5 Department of Respiratory Medicine, Mater Health Services, South Brisbane, QLD, Australia; 6 Mater Research Institute, University of Queensland, Brisbane, Australia; 7 Department of Respiratory Diseases, Aalborg University Hospital, Aalborg, Denmark; 8 CEMER Respiratory Research, Vicente López, Buenos Aires, Argentina; 9 Late-Stage Respiratory & Immunology, BioPharmaceuticals R&D, AstraZeneca, Gothenburg, Sweden; 10 Late-Stage Respiratory & Immunology, BioPharmaceuticals R&D, AstraZeneca, Gaithersburg, MD, USA; 11 Late-Stage Respiratory & Immunology, BioPharmaceuticals R&D, AstraZeneca, Barcelona, Spain; 12 Translational Science and Experimental Medicine, Early Respiratory & Immunology, BioPharmaceuticals R&D, AstraZeneca, Gaithersburg, MD, USA; 13 Translational Science and Experimental Medicine, Early Respiratory & Immunology, BioPharmaceuticals R&D, AstraZeneca, Gothenburg, Sweden

## Abstract

**Background:**

Bronchiectasis is a chronic, inflammatory lung disease with distinct endotypes, often defined by inflammatory cells in blood and sputum. We assessed safety and efficacy of benralizumab, an anti-interleukin-5Rα monoclonal antibody, in patients with bronchiectasis and eosinophilic inflammation.

**Methods:**

MAHALE (NCT05006573) was a 52-week, randomised, multicentre, double-blind, phase 3 study. Patients with bronchiectasis and no comorbid pulmonary conditions were randomised 1:1 to receive subcutaneous benralizumab 30 mg or placebo every 4 weeks. Primary end-point was annualised exacerbation rate. Secondary end-points included: time to first exacerbation, change from baseline in respiratory symptoms, lung function, health-related quality of life (HRQoL), respiratory health status and safety. In August 2022, the study was terminated early due to recruitment challenges related to the COVID-19 pandemic.

**Results:**

100 patients were randomised and 99 were dosed (benralizumab, n=54; placebo, n=45); 21 had blood eosinophil counts ≥300 cells·µL^−1^. Notable imbalances in baseline characteristics between treatment arms suggested patients receiving benralizumab had more severe disease. The study did not enrol enough patients to meet power requirement to detect differences in efficacy end-points between benralizumab and placebo. At the end of the study, annualised exacerbation rates were not significantly different between treatment groups (rate ratio (95% confidence interval): 1.14 (0.71–1.82), p=0.59). Change in respiratory symptoms and HRQoL were similar between treatment groups. Benralizumab was well tolerated.

**Conclusions:**

A small sample size with eosinophil counts predominantly <300 cells·µL^−1^ and imbalances at baseline limit interpretation of data on clinical outcomes following benralizumab treatment. Adequately powered randomised trials are needed in the population of interest.

## Shareable abstract

**In MAHALE, benralizumab did not improve bronchiectasis clinical outcomes. Adequately powered randomised trials are needed in the population of interest.** https://bit.ly/4dFjR40

## Introduction

Bronchiectasis is a chronic, inflammatory lung disease encompassing a wide range of aetiologies and is estimated to affect up to 566 per 100 000 females and 485.5 per 100 000 males; although awareness has increased over time, underdiagnosis and diagnostic delays still exist [1–3]. The disease is characterised by permanent airway dilatation and impaired mucociliary clearance, leading to chronic productive cough, recurrent respiratory infections and persistent daily symptoms of sputum production, breathlessness and fatigue [4–8]. Daily symptoms are a major determinant of quality of life (QoL) [9], and many patients experience limitations to their daily activities [10]. Symptom burden is also predictive of future exacerbation risk [11]. Exacerbations are a key driver of disease progression [11], and studies consistently show that patients with frequent exacerbations experience poor long-term clinical outcomes [7, 12]. Consequently, the goals of bronchiectasis treatment are to prevent exacerbations, alleviate symptoms, improve QoL and prevent disease progression [11].

Though historically considered a neutrophilic disease, bronchiectasis is increasingly recognised as a complex condition with distinct endotypes, often defined by the predominance of specific inflammatory cell types in blood or sputum [13–16]. This type of treatable traits framework may better address heterogeneous airways diseases by enabling more targeted, personalised treatment approaches [17]. For example, targeting specific inflammatory cells or pathways may help ameliorate symptoms and reduce exacerbations and hospitalisations of patients with bronchiectasis with the same specific endotype. Despite the increasing recognition of distinct bronchiectasis endotypes, few evidence-based, targeted therapies currently exist [4, 18], underscoring the urgent need for novel treatment approaches tailored to specific endotypes.

An eosinophilic endotype of bronchiectasis has been described by Shoemark and colleagues [15], based on the presence of elevated blood eosinophils (bEOS) related to sputum eosinophilic inflammation, airway infection with *Pseudomonas aeruginosa* and increased risk of exacerbations. Elevated bEOS counts (≥300 cells·µL^−1^) have been shown to be associated with an increased risk of bronchiectasis exacerbations and hospitalisations [15, 19]. ∼17–30% of patients with bronchiectasis have elevated bEOS counts and may therefore benefit from treatments that deplete eosinophils [14, 16, 20]. Elevated bEOS are evident even after the careful exclusion of patients with asthma and allergic bronchopulmonary aspergillosis (conditions that could cause increased bEOS), and are an indicator of a distinct endotype of bronchiectasis; however, there are limited studies on eosinophilic bronchiectasis [20].

Benralizumab is a humanised afucosylated anti-interleukin-5 receptor α monoclonal antibody causing near complete depletion of eosinophils *via* antibody-dependent cellular cytotoxicity [21, 22]. Benralizumab has demonstrated efficacy in, and has been approved for the treatment of, severe eosinophilic asthma [23–25] and eosinophilic granulomatosis with polyangiitis [24–26]. A case series of 21 patients with bronchiectasis and eosinophil infiltration has reported that benralizumab or mepolizumab (an anti-interleukin-5 monoclonal antibody) could reduce bEOS and improve health-related quality of life (HRQoL) [14]; however, this study was not controlled and included patients with overlapping eosinophil-associated diseases.

Here, we report results from the MAHALE study, a phase 3, randomised, double-blind (DB) clinical trial designed to evaluate the suitability of benralizumab for the treatment of patients with bronchiectasis with an eosinophilic endotype and no other respiratory comorbidities (*e.g.*, asthma or COPD).

## Methods

### Study design and interventions

MAHALE (NCT05006573) was a 52-week, randomised, multicentre, DB, parallel-group, placebo-controlled, phase 3 study to evaluate the safety and efficacy of benralizumab *versus* placebo when administered in addition to standard-of-care therapy in adults with bronchiectasis and eosinophilic inflammation.

The study was conducted across 15 countries: Argentina, Australia, Canada, China, Denmark, Germany, India, Italy, Philippines, Poland, Russia, South Korea, Spain, Vietnam and the USA. The study was initiated in July 2021 and was terminated early in August 2022 due to recruitment challenges related to the COVID-19 pandemic. The study was not terminated due to safety or efficacy concerns.

The study was conducted in accordance with the Declaration of Helsinki and Council for International Organizations of Medical Sciences International Ethical Guidelines, and the International Conference on Harmonization GCP Guidelines. The protocol, protocol amendments and any other relevant documents were reviewed and approved by an independent ethics committee. Written informed consent was obtained for all patients. The trial is registered with ClinicalTrials.gov, where the study protocol is also available (https://clinicaltrials.gov/study/NCT05006573). Patients were stratified by country, long-term macrolide use and bEOS count. The cut-off to define eosinophilic endotype was ≥300 cells·µL^−1^, based on the work by Shoemark and colleagues [15]. A cohort of patients with bEOS <300 cells·µL^−1^ was included to determine if any benefit could be observed in this group, given the limited data available on this eosinophilic endotype at the time of study design. Patients had two tests for bEOS count during screening.

**FIGURE 1:**
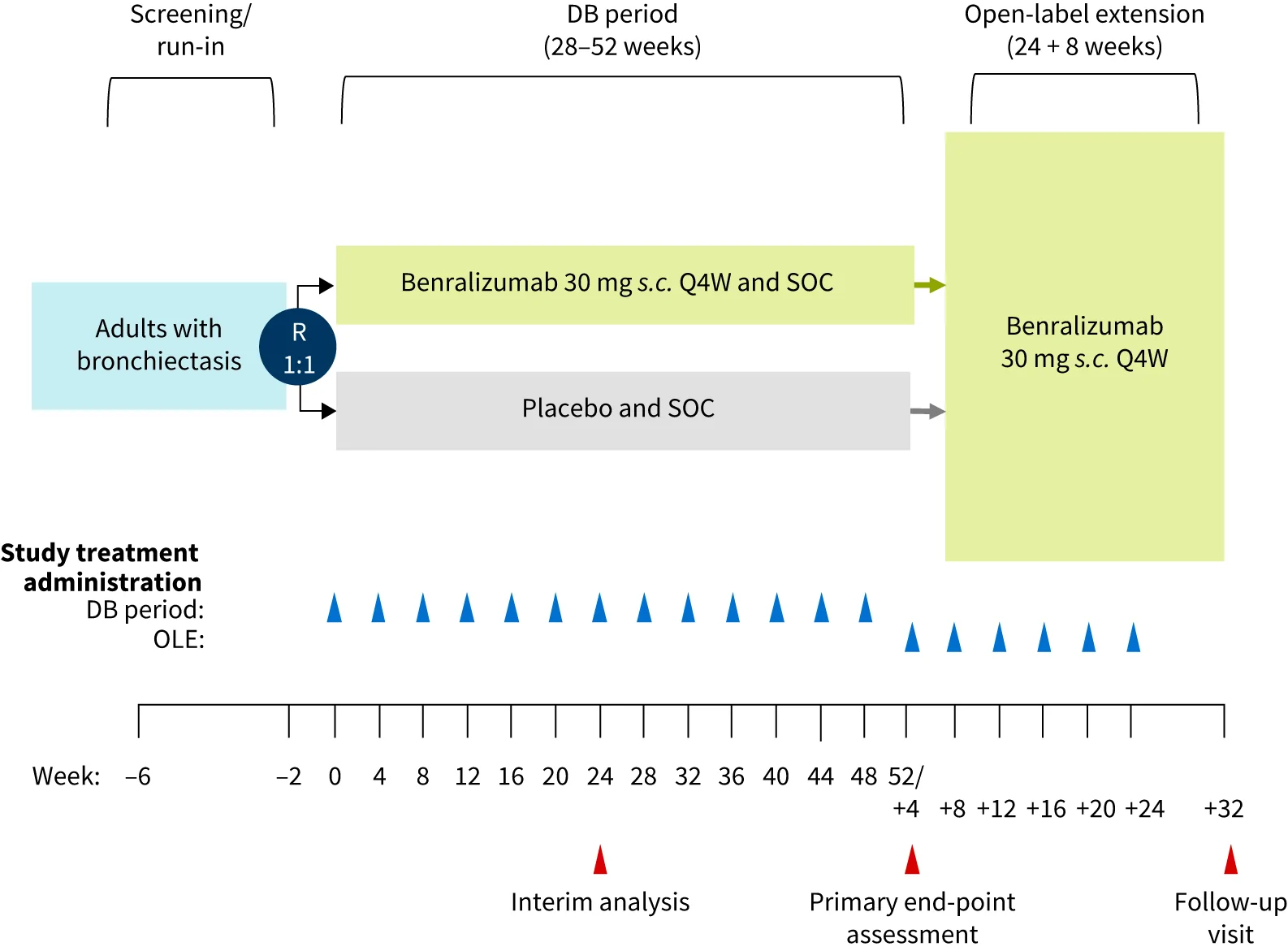
Study design. Patients could continue into the OLE period once they had completed at least 28 weeks of the DB period. DB: double-blind; OLE: open-label extension; Q4W: every 4 weeks; R: randomisation; *s.c.*: subcutaneous; SOC: standard of care.

Patients were randomised 1:1 to receive subcutaneous benralizumab 30 mg or placebo every 4 weeks (figure 1). The original duration of the DB period was 52 weeks; however, after a protocol amendment was implemented to terminate the study in January 2023, patients who had already been randomised were allowed to continue treatment in the DB period for at least 28 weeks, leading to a DB period duration ranging from 28 to 52 weeks. Once they had completed at least 28 weeks of the DB period, all patients were allowed to continue into an open-label extension (OLE) period of 24 weeks in which they received benralizumab 30 mg, with a follow-up visit 8 weeks after the last dose of study treatment.

Feedback on the study design was obtained from a panel of 10 patients with bronchiectasis who participated in interviews conducted over 3 days in June 2020. Based on their input, the study protocol was amended to include optional self-administration of the study treatment, allowance for the use of historical CT scans for eligibility assessment and a reduced frequency of selected study assessments.

### Patient population

The patient population comprised individuals with a diagnosis of bronchiectasis confirmed by computerised tomography scan (>1 segment affected within a single lobe), ≥2 bronchiectasis exacerbations in the past year and in receipt of airway clearance therapy, physiotherapy or mucus clearance therapy (dose and regimen of any drugs used needed to be stable for ≥3 months prior to screening and remain stable throughout the DB period). Patients with pulmonary disease other than bronchiectasis, including COPD, asthma, cystic fibrosis, respiratory infection or any other disease associated with elevated bEOS count, were excluded on clinical grounds. A full list of eligibility criteria is available in the supplementary methods.

### End-points

The primary end-point was annualised bronchiectasis exacerbation rate. Bronchiectasis exacerbations were defined using the European Multicentre Bronchiectasis Audit and Research Collaboration/US Bronchiectasis Research Registry/COPD Foundation (EMBARC/BRR) definition [8] as deterioration in ≥3 key symptoms for at least 48 h with a treating physician determining that a change in treatment (*e.g.*, initiation of a new antibiotic medication) was required, and/or hospitalisation or death due to bronchiectasis. Key symptoms were cough, sputum volume and/or consistency, sputum purulence, breathlessness and/or exercise intolerance, fatigue and/or malaise, and haemoptysis.

Secondary end-points were time to first bronchiectasis exacerbation, change from baseline in respiratory symptoms assessed by the QoL Questionnaire – Bronchiectasis Respiratory Symptom Scale (QoL-B-RSS), and change from baseline in lung function (pre-bronchodilator (BD) forced expiratory volume in 1 s (FEV_1_)). Additional secondary end-points were change from baseline in HRQoL (Quality of Life – Bronchiectasis (QoL-B), St George's Respiratory Questionnaire (SGRQ) and Leicester Cough Questionnaire (LCQ)) and safety (including adverse events (AEs)). Change from baseline in eosinophils in blood and sputum were exploratory end-points. The OLE assessed safety, concomitant medication use and exacerbations; no efficacy evaluations occurred after the final visit of the DB treatment period except for exacerbations.

The full analysis set (FAS) comprised randomised patients who received at least one dose of study treatment.

### Statistical analyses and sample size calculation

In the initial protocol, ∼420 patients were to be randomised. Assuming an annual exacerbation rate of 1.5 events per patient per year, it was estimated that 280 patients should be randomised into the ≥300 cells·µL^−1^ strata (n=140 in each treatment group) to achieve a >90% power of detecting a 40% reduction in exacerbation rate with benralizumab *versus* placebo, using a two-sided 5% α level test. An approximate 2:1 ratio was planned in the ≥300 cells·µL^−1^ and <300 cells·µL^−1^ bEOS strata, meaning 140 patients were to be randomised into the <300 cells·µL^−1^ strata (n=70 in each treatment group). However, due to premature termination of enrolment into the study, sample sizes (overall and within strata) were smaller than planned. Consequently, the primary efficacy analysis was conducted in the FAS (*i.e.*, regardless of bEOS strata) and the study was not powered to assess the primary efficacy end-point.

The annualised bronchiectasis exacerbation rate in the treatment groups was compared using a negative binomial model, with response variables including the number of bronchiectasis exacerbations experienced by a patient over the 52-week treatment period and covariates of treatment group, number of exacerbations from previous year, long-term macrolide use (yes/no), *P. aeruginosa* sputum culture status (yes/no) and region. Patients’ variable treatment periods were accounted for in the model. The log of follow-up time was included in the negative binomial model as an offset to account for this. Time to first exacerbation was analysed using a Cox proportional hazards model fitted to the data with covariates of treatment group, long-term macrolide use (yes/no), *P. aeruginosa* sputum culture status (yes/no), number of exacerbations in previous year and region. Change from baseline in pre-BD FEV_1_ and HRQoL were compared between treatment groups using a mixed-effect model for repeated measures analysis.

## Results

### Disposition and baseline characteristics

**FIGURE 2:**
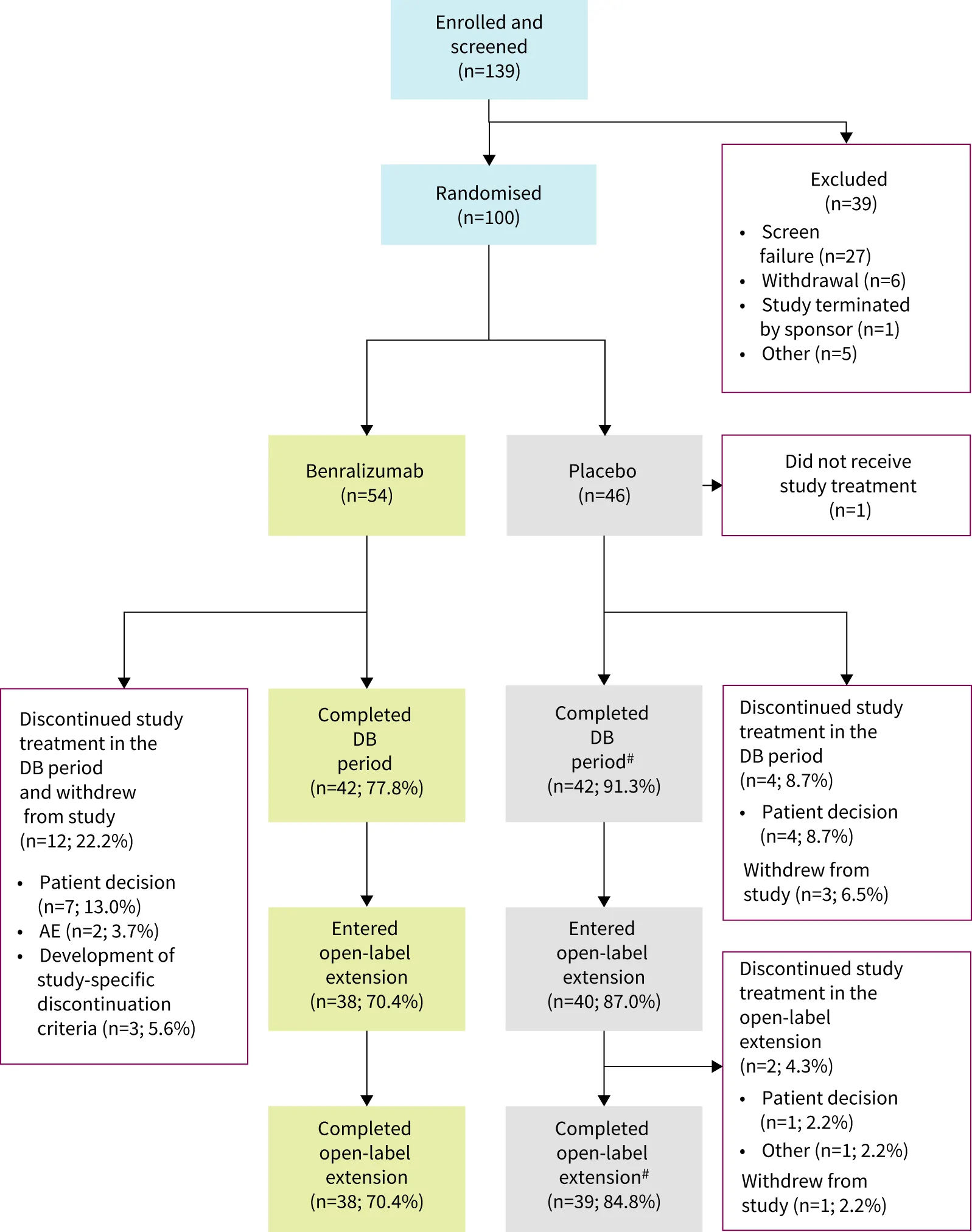
Study disposition. ^#^: includes patients who discontinued study treatment but attended all study visits. DB: double-blind; AE: adverse event.

At the time when recruitment was stopped, 139 patients had been screened, of which 100 were randomised and 99 were dosed (benralizumab, n=54; placebo, n=45). One patient (placebo group) did not receive study medication (figure 2).

Of the 99 randomised patients who received study treatment, 21 had a baseline bEOS count ≥300 cells·µL^−1^ and 78 had a baseline bEOS count <300 cells·µL^−1^. Mean baseline beEOS counts are shown in supplementary figure S1.

There were notable differences between treatment arms in demographics and disease characteristics, suggesting that patients receiving benralizumab had more severe disease at baseline (table 1; supplementary tables S1–S6). Compared with the placebo group, the benralizumab group had proportionally fewer females, more patients with a *P. aeruginosa* positive culture, longer disease duration (time since symptoms and diagnosis) and more severe disease (based on lower QoL-B scores, higher SGRQ score, more patients with a severe bronchiectasis severity index score, greater use of mucolytics and manual chest physical therapy, and lower pre-BD FEV_1_). When analysed by subgroups, further imbalances were observed (supplementary tables S2‒6), with higher proportions of some severe subgroups in the benralizumab arm (table 1). Patients who were *P. aeruginosa* positive had a higher level of disease severity *versus* those who were *P. aeruginosa* negative; however, prior exacerbation rates were similar between the two subgroups (supplementary table S4).

**TABLE 1:** Baseline demographics and characteristics of patients who were randomised and received study treatment

Baseline demographics/characteristics	Benralizumab 30 mg	Placebo	Total
**Patients, n**	54	45	99
**Age years, mean±sd**	59.2±13.1	59.2±15.5	59.2±14.2
**Sex, n (%)**			
Male	17 (31.5)	10 (22.2)	27 (27.3)
Female	37 (68.5)	35 (77.8)	72 (72.7)
**Race, n (%)**			
White	36 (66.7)	31 (68.9)	67 (67.7)
Asian	17 (31.5)	14 (31.1)	31 (31.3)
Other	1 (1.9)	0 (0)	1 (1.0)
**Region, n (%)**			
North America	4 (7.4)	4 (8.9)	8 (8.1)
Europe	24 (44.4)	17 (37.8)	41 (41.4)
Asia	15 (27.8)	13 (28.9)	28 (28.3)
Other (Argentina/Australia)	11 (20.4)	11 (24.4)	22 (22.2)
**Blood eosinophil count cells·µL^−1^**			
Mean±sd	211±214.0	204±211.4	208±211.8
Median (range)	150 (30–1030)	130 (10–1260)	140 (10–1260)
<300 cells·µL^−1^, n (%)	42 (77.8)	36 (80.0)	78 (78.8)
≥300 cells·µL^−1^, n (%)	12 (22.2)	9 (20.0)	21 (21.2)
***Pseudomonas aeruginosa* status, n (%)**			
Positive	19 (38.0)^#^	6 (14.6)^¶^	25 (27.5)^+^
Negative	31 (62.0)^#^	35 (85.4)^¶^	66 (72.5)^+^
Missing, n	4	4	8
**History of positive sputum culture, n (%)**	35 (64.8)	21 (46.7)	56 (56.6)
**Nonsmokers, n (%)**	42 (77.8)	36 (80.0)	78 (78.8)
**Time since first bronchiectasis symptoms years, mean±sd**	23.3±21.5^§^	14.7±17.1	19.3±19.9^ƒ^
**Time since bronchiectasis diagnosis years, mean±sd**	18.2±19.7	12.9±17.2	15.8±18.7
**Number of prior exacerbations, mean±sd**	2.6±1.0	2.6±0.9	2.6±1.0
**Treatments, n (%)**			
Prophylactic macrolides ≥3 months	7 (13.0)	5 (11.1)	12 (12.1)
Maintenance ICS	25 (46.3)	20 (44.4)	45 (45.5)
Manual chest physical therapy	33 (61.1)	22 (48.9)	55 (55.6)
Positive expiratory pressure device	13 (24.1)	9 (20.0)	22 (22.2)
Chest wall oscillation vest	2 (3.7)	3 (6.7)	5 (5.1)
Nebulisation	15 (27.8)	10 (22.2)	25 (25.3)
Mucolytic	24 (44.4)	10 (22.2)	34 (34.3)
Other	18 (33.3)	18 (40.0)	36 (36.4)
**Pre-BD FEV_1_ L, mean±sd**	1.75±0.68^§^	1.96±0.68^##^	1.85±0.69^¶¶^
**Pre-BD FEV_1_ % predicted, mean±sd**	64.8±25.7^§^	74.4±22.9^##^	69.2±24.8^¶¶^
**QoL-B respiratory symptom score, mean±sd^++^**	54.7±16.5	65.1±16.6^##^	59.4±17.2^§§^
**LCQ score, mean±sd^ƒƒ^**	13.8±3.7	14.6±4.0^##^	14.2±3.9^§§^
**SGRQ score, mean±sd^###^**	48.5±19.1^¶¶¶^	39.7±22.7^+++^	44.8±21.0^§§§^
**BSI score, males, mean±sd**	10.1±6.0^ƒƒƒ^	7.2±5.4^####^	9.4±5.9^¶¶¶¶^
**BSI score, females, mean±sd**	7.4±4.4^++++^	7.0±3.4^§§§§^	7.2±3.9^ƒƒƒƒ^
**BSI category, n (%)^#####^**			
Mild (BSI ≤4)	11 (25.6)^¶¶¶¶¶^	8 (25.8)^+++++^	19 (25.7)^§§§§§^
Moderate (BSI 5–8)	15 (34.9)^¶¶¶¶¶^	15 (48.4)^+++++^	30 (40.5)^§§§§§^
Severe (BSI >8)	17 (39.5)^¶¶¶¶¶^	8 (25.8)^+++++^	25 (33.8)^§§§§§^

Baseline refers to the value at randomisation visit or, if missing, the closest value prior to randomisation. ICS: inhaled corticosteroids; BD: bronchodilator; FEV_1_: forced expiratory volume in 1 s; QoL-B: quality of life – bronchiectasis; LCQ: Leicester Cough Questionnaire; SGRQ: St George's Respiratory Questionnaire; BSI: bronchiectasis severity index; HRQoL: health-related quality of life. ^#^: n=50; ^¶^: n=41; ^+^: n=91; ^§^: n=53; ^ƒ^: n=98; ^##^: n=44; ^¶¶^: n=97; ^++^: QoL-B is assessed on a scale of 0–100 (lower scores indicate worse HRQoL, higher scores indicate better HRQoL); ^§§^: n=98; ^ƒƒ^: LCQ is assessed on a scale of 3–21 (lower scores indicate worse HRQoL, higher scores indicate better HRQoL); ^###^: SGRQ is assessed on a scale of 0–100 (lower scores indicate better HRQoL, higher scores indicate worse HRQoL); ^¶¶¶^: n=52; ^+++^: n=37; ^§§§^: n=89; ^ƒƒƒ^: n=15; ^####^: n=5; ^¶¶¶¶^: n=20; ^++++^: n=28; ^§§§§^: n=26; ^ƒƒƒƒ^: n=54; ^#####^: BSI is assessed on a scale of 0–26 (≤4=mild bronchiectasis; 5–8=moderate bronchiectasis; >8=severe bronchiectasis); ^¶¶¶¶¶^: n=43; ^+++++^: n=31; ^§§§§§^: n=74.

### Primary end-point (annualised exacerbation rate)

Annualised exacerbation rates were not significantly different between benralizumab and placebo (rate ratio 1.14, 95% confidence interval (CI): 0.71–1.82, p=0.591) (table 2). Crude exacerbation rates were lower in the OLE period *versus* the DB period for benralizumab and placebo (table 2). Due to the recruitment challenges listed earlier, the study was not adequately powered to demonstrate differences between treatment groups.

**TABLE 2:** Annualised rate of bronchiectasis exacerbations

	Overall		Blood eosinophils <300 cells·µL^−1^		Blood eosinophils ≥300 cells·µL^−1^	
	Benralizumab 30 mg	Placebo	Benralizumab 30 mg	Placebo	Benralizumab 30 mg	Placebo
**DB period, n**	54	45	42	36	12	9
Total number of exacerbations	67	52	55	43	12	9
Total follow-up time years	47.3	41.3	36.4	32.7	10.9	8.6
Crude rate	1.4	1.3	–	–	–	–
Annualised exacerbation rate (95% CI)	1.44 (1.05–1.97)	1.27 (0.89–1.80)	1.53 (1.08–2.17)	1.32 (0.89–1.94)	1.12 (0.55–2.27)	1.08 (0.48–2.44)
Rate ratio (95% CI), p-value	1.14 (0.71–1.82), 0.591		1.16 (0.69–1.96), 0.573		1.04 (0.35–3.06), 0.945	
Difference in rates (95% CI)	0.17 (−0.46–0.81)		0.21 (−0.53–0.95)		0.04 (−1.14–1.23)	
**OLE period, n^#^**	38	40	–	–	–	–
Total number of exacerbations	15	12	–	–	–	–
Total follow-up time years	23.6	24.2	–	–	–	–
Crude rate	0.6	0.5	–	–	–	–

Annualised exacerbation rate was calculated by the number of exacerbations ×365.25/total follow-up time within the DB period in days. DB: double-blind; OLE: open-label extension. ^#^: all patients were receiving active drug during the OLE period.

### Secondary end-points (time to first exacerbation, QoL-B-RSS, pre-BD FEV_1_)

**FIGURE 3:**
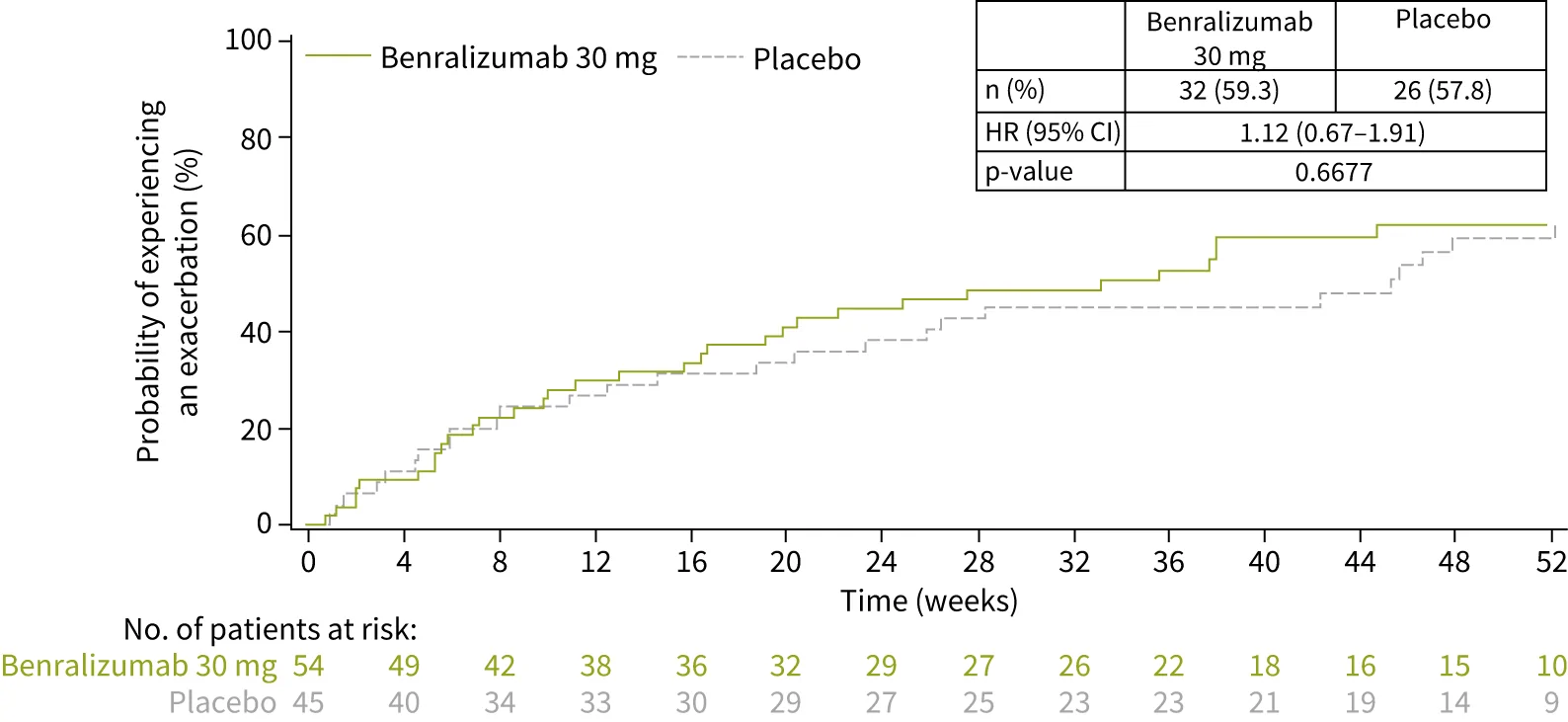
Probability of a patient experiencing an exacerbation during the 52-week double-blind period. HR: hazard ratio.

The median time to first exacerbation was 233 days in the benralizumab group and 316 days in the placebo group (hazard ratio 1.12, 95% CI: 0.67–1.91, p=0.6677) (figure 3). Exacerbations occurred in 32 (59.3%) patients in the benralizumab group and 26 patients (57.8%) in the placebo group.

**FIGURE 4:**
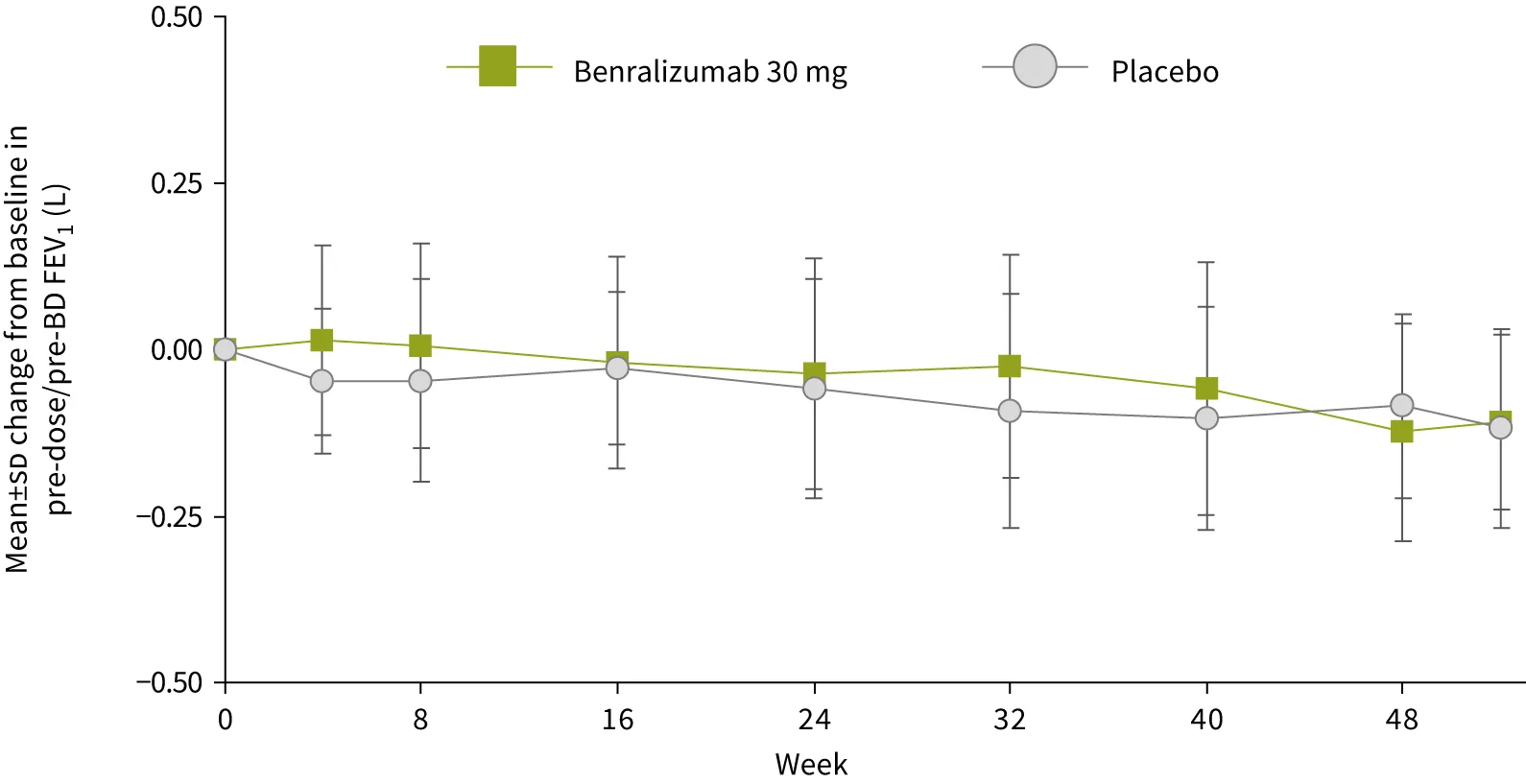
Change from baseline in pre-BD FEV_1_. BD: bronchodilator; FEV_1_: forced expiratory volume in 1 s.

The change from baseline in pre-BD FEV_1_ was similar in the two treatment groups (figure 4).

### Other secondary end-points

**FIGURE 5:**
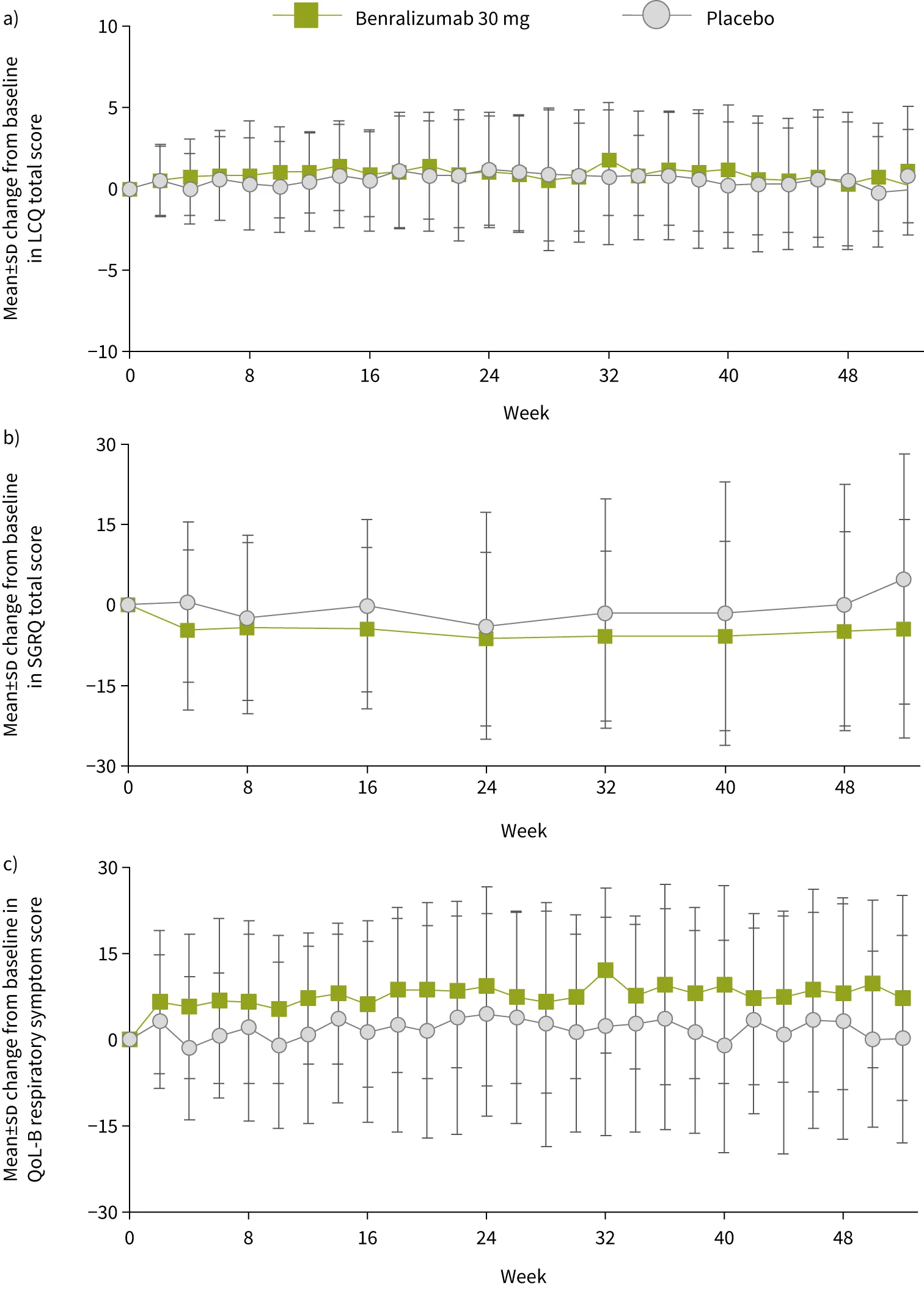
Change from baseline in a) Leicester Cough Questionnaire (LCQ), b) St George's Respiratory Questionnaire (SGRQ) and c) Quality of Life – Bronchiectasis Respiratory Symptom Scale (QoL-B RSS) from Week 0 to 52. a) LCQ is assessed on a scale of 3–21 (lower scores indicate worse health-related quality of life (HRQoL), higher scores indicate better HRQoL); b) SGRQ is assessed on a scale of 0–100 (lower scores indicate better HRQoL, higher scores indicate worse HRQoL); c) QoL-B is assessed on a scale of 0–100 (lower scores indicate worse HRQoL, higher scores indicate better HRQoL; minimal clinically important differences for respiratory symptom score=8) [9, 41].

The change from baseline in LCQ, SGRQ and QoL-B respiratory symptoms and other QoL-B domains were similar between the two treatment groups (figure 5 and supplementary figure S2).

### Pharmacodynamic effect of benralizumab (exploratory end-point)

Benralizumab demonstrated a rapid, near complete and sustained depletion of bEOS compared with placebo (supplementary figure S3). Benralizumab also demonstrated a local pharmacodynamic effect, as eosinophils in sputum were depleted with benralizumab treatment during the DB period; however, the number of patients with sputum eosinophil data was small (supplementary figure S4).

### Safety

A total of 88.9% of patients in the benralizumab group and 73.3% of patients in the placebo group experienced AEs during the DB period of the study. The most common AEs during the DB period were COVID-19 (benralizumab, 33.3%; placebo, 22.2%), bronchiectasis (benralizumab, 14.8%; placebo, 6.7%) and nasopharyngitis (benralizumab, 9.3%; placebo, 11.1%). Overall, 18.5% and 6.7% of patients in the benralizumab and placebo groups, respectively, experienced a serious AE (SAE) (table 3). Based on exposure-adjusted rates, the incidence rate per 100 patient-years was 101.2 AEs and 21.1 SAEs in the benralizumab group, and 79.6 AEs and 7.2 SAEs in the placebo group. All SAEs were resolved by the time of database lock, and none were assessed by the investigator to be related to the study treatment (supplementary tables S7 and S8). Two patients in the benralizumab group experienced AEs that led to treatment discontinuation (pneumonia (n=1) and bronchiectasis (n=1)). There were no deaths in the study and no new safety concerns were identified.

**TABLE 3:** Incidence of adverse events (AEs)

Preferred term	Benralizumab 30 mg^#^		Placebo^¶^		Total while on benralizumab 30 mg (including OLE)^+^	
	n (%)	EAR^§^	n (%)	EAR^§^	n (%)	EAR^§^
**Any AE**	48 (88.9)	101.2	33 (73.3)	79.6	67 (71.3)	70.4
Leading to discontinuation of treatment	2 (3.7)	–	0 (0)	–	2 (2.1)	–
Leading to death	0 (0)	–	0 (0)	–	0 (0)	–
**Any SAE**	10 (18.5)	21.1	3 (6.7)	7.2	17 (18.1)	17.9
**Most frequent AEs (>5% in any group) by MedDRA preferred term**						
COVID-19	18 (33.3)	37.9	10 (22.2)	24.1	23 (24.5)	24.2
Bronchiectasis	8 (14.8)	16.9	3 (6.7)	7.2	12 (12.8)	12.6
Nasopharyngitis	5 (9.3)	10.5	5 (11.1)	12.1	7 (7.4)	7.4
Headache	5 (9.3)	10.5	5 (11.1)	12.1	7 (7.4)	7.4
Back pain	4 (7.4)	8.4	2 (4.4)	4.8	5 (5.3)	5.3
GERD	4 (7.4)	8.4	0 (0)	–	4 (4.3)	4.2
Sinusitis	3 (5.6)	6.3	1 (2.2)	2.4	6 (6.4)	6.3
Upper respiratory tract infection	3 (5.6)	6.3	1 (2.2)	2.4	5 (5.3)	5.3
UTI	3 (5.6)	6.3	0 (0)	–	3 (3.2)	3.2
Upper abdominal pain	3 (5.6)	6.3	0 (0)	–	3 (3.2)	3.2
Fatigue	3 (5.6)	6.3	0 (0)	–	4 (4.3)	4.2

MedDRA version 26.1. OLE: open-label extension; EAR: exposure-adjusted rate; SAE: serious adverse event; MedDRA: Medical Dictionary for Regulatory Activities; GERD: gastroesophageal reflux disease; UTI: urinary tract infection. ^#^: n=54; ^¶^: n=45; ^+^: n=94; ^§^: per 100 patient-years.

## Discussion

The MAHALE trial was terminated early due to recruitment challenges during the COVID-19 pandemic. Due to small sample size, the study was underpowered to detect differences in efficacy end-points. The primary efficacy analysis was conducted in the FAS, in which the majority of patients had a bEOS count <300 cells·µL^−1^. Therefore, care must be taken in interpreting the results of this study, as the planned enrolment for statistical power was not met.

Elevated bEOS counts (≥300 cells·µL^−1^) are associated with an increased risk of exacerbations [19]. Targeting type 2 inflammatory pathways, including eosinophil depletion, was hypothesised to reduce exacerbations and improve daily symptoms by attenuating inflammation in patients with an eosinophilic endotype. In this study, depletion of eosinophils with benralizumab was consistent with previous observations, confirming its expected mechanism of action [14, 27]. Despite the near complete depletion of blood and sputum eosinophils by benralizumab, there was no observed reduction of exacerbations, or improvement in respiratory function or HRQoL measures compared with placebo. However, the small number of patients randomised, the limited target population and the imbalances between treatment groups at baseline constrain the interpretation of these efficacy data. Previous case series involving benralizumab and mepolizumab in patients with bronchiectasis reported reductions in bEOS, improvements in lung function, reduced exacerbation rates and enhancements in HRQoL, alongside a potential oral corticosteroid-sparing effect [14, 28]. However, those studies lacked control groups and included patients with overlapping eosinophilic conditions, limiting the generalisability of the findings. Most importantly, data supporting the eosinophilic endotype in bronchiectasis currently suggest the target population for anti-eosinophil therapy would be patients with bEOS ≥300 cells·µL^−1^ [14, 15, 19, 20]. Only 21 patients with bEOS ≥300 cells·µL^−1^ were recruited into this study, so no conclusions on the target population can be drawn. Patients with bEOS <300 cells·µL^−1^ were included to better understand the role of eosinophils in bronchiectasis; this threshold was chosen because the optimal threshold for defining the eosinophilic endotype of bronchiectasis was not established at the time the study was designed. Based on the clinical data to date, it is unclear whether eosinophils are directly involved in disease pathogenesis or are bystanders. The results observed in this study could also reflect the limitations of elevated bEOS as a biomarker of eosinophil activity, rather than suggest eosinophils are not involved in the underlying cause of the disease. This study may thus aid the design of further clinical trials, which would be required to answer these questions and to establish if targeting eosinophil depletion with benralizumab or other anti-eosinophil therapies has benefits in patients with bronchiectasis of an eosinophilic endotype. Another factor limiting the interpretation of this study is that there were notable differences in baseline characteristics between groups, which may have impacted the efficacy and safety observations. Lower HRQoL, more patients with *P. aeruginosa*-positive cultures and longer disease duration may indicate more severe disease and/or worse symptoms in the benralizumab arm at baseline. Sex is known to be associated with differences in disease severity, symptoms, lung function and HRQoL in airway diseases such as bronchiectasis [29–31]; therefore, disparities in the number of male and female patients may impact imbalances at baseline.

There were some numerical disparities in AEs and SAEs reported during the study, which may also reflect a greater disease severity at baseline in the benralizumab group. Chronic *P. aeruginosa* infection has been shown to be independently associated with frequency of bronchiectasis exacerbations, hospital admissions, worse HRQoL and increased mortality, particularly in patients who experienced frequent exacerbations (≥2 per year) [32]. The higher incidence of *P. aeruginosa-*positive patients in the benralizumab group compared with the placebo group may have contributed to these differences in AEs and SAEs. Nevertheless, treatment with benralizumab was well tolerated in patients with bronchiectasis, with no new safety concerns. Results from the study were generally consistent with the known safety profile of benralizumab in other diseases [23, 26, 33–35].

Exacerbation rates during the OLE were much lower than in the DB period, highlighting the value of DB studies and the need for caution when interpreting efficacy findings from unblinded treatment studies. The lower exacerbation rates during the OLE could also be due to “regression to the mean”, where patients who experience a high number of exacerbations during one period are likely to experience fewer exacerbations in subsequent periods due to normal variability [36]. Care must be taken when interpreting the results of patient-reported outcome assessments; although the psychometric properties of LCQ [37] and SGRQ [38] in bronchiectasis have been explored, content validity evidence is lacking. More research is needed to establish whether these questionnaires meaningfully cover the respiratory symptoms and HRQoL impacts that matter to patients with bronchiectasis.

Given the imbalances between treatment arms in disease characteristics at baseline, future studies should consider stratifying patients based on sex, disease severity and *P. aeruginosa* status, and have a more enriched primary population. Finally, strict exclusion of patients with other airway diseases, including where these were considered secondary diagnoses, made recruitment challenging, as it is reported that 30% to 68% of patients with bronchiectasis have comorbid asthma or COPD [39]. Recent data from the EMBARC registry suggests that the label of COPD is often applied to patients with bronchiectasis when there is no objective evidence of COPD [40], and this may lead to inappropriate exclusion of some patients from bronchiectasis trials. Asthma diagnoses are similarly challenging in this population, due to the lack of a single, definitive and accurate diagnostic test. Future clinical trials in patients with bronchiectasis should ensure that the proportions of patients with comorbid pulmonary conditions are not over-represented in the study population, so that proper evaluation of bronchiectasis-specific treatment effects can be observed.

In conclusion, benralizumab achieved its systemic pharmacodynamic effect by depleting bEOS, and also demonstrated a local pharmacodynamic effect by depleting sputum eosinophils in the limited number of patients for whom these data were available. However, treatment with benralizumab was not associated with improved clinical outcomes in patients with bronchiectasis but was well tolerated with no new safety concerns other than those described in other studies. Due to the small sample size, imbalances between treatment groups at baseline and the enrolment of a predominantly noneosinophilic population, these findings should be considered hypothesis generating and need to be confirmed in appropriately powered randomised controlled trials. Accordingly, these findings should not be interpreted as excluding a potential role for eosinophil-targeting therapies in appropriately selected patients with bronchiectasis.

## Data Availability

statement: Data underlying the findings described in this manuscript may be obtained in accordance with AstraZeneca's data sharing policy described at https://www.astrazenecaclinicaltrials.com/our-transparency-commitments/
